# The Correlation Between Wall Shear Stress and Plaque Composition in Advanced Human Carotid Atherosclerosis

**DOI:** 10.3389/fbioe.2021.828577

**Published:** 2022-01-28

**Authors:** A. M. Moerman, S. Korteland, K. Dilba, K. van Gaalen, D. H. J. Poot, A. van Der Lugt, H. J. M. Verhagen, J. J. Wentzel, A. F. W. van Der Steen, F. J. H. Gijsen, K. Van der Heiden

**Affiliations:** ^1^ Department of Biomedical Engineering, Thorax Center, Erasmus MC, Rotterdam, Netherlands; ^2^ Department of Radiology and Nuclear Medicine, Erasmus MC, Rotterdam, Netherlands; ^3^ Department of Vascular Surgery, Erasmus MC, Rotterdam, Netherlands; ^4^ Department of Biomedical Engineering, Delft University of Technology, Delft, Netherlands

**Keywords:** atherosclerosis, wall shear stress, oscillatory shear index, vulnerable plaque, plaque composition, MRI, image registration pipeline

## Abstract

The role of wall shear stress (WSS) in atherosclerotic plaque development is evident, but the relation between WSS and plaque composition in advanced atherosclerosis, potentially resulting in plaque destabilization, is a topic of discussion. Using our previously developed image registration pipeline, we investigated the relation between two WSS metrics, time-averaged WSS (TAWSS) and the oscillatory shear index (OSI), and the local histologically determined plaque composition in a set of advanced human carotid plaques. Our dataset of 11 carotid endarterectomy samples yielded 87 histological cross-sections, which yielded 511 radial bins for analysis. Both TAWSS and OSI values were subdivided into patient-specific low, mid, and high tertiles. This cross-sectional study shows that necrotic core (NC) size and macrophage area are significantly larger in areas exposed to high TAWSS or low OSI. Local TAWSS and OSI tertile values were generally inversely related, as described in the literature, but other combinations were also found. Investigating the relation between plaque vulnerability features and different combinations of TAWSS and OSI tertile values revealed a significantly larger cap thickness in areas exposed to both low TAWSS and low OSI. In conclusion, our study confirmed previous findings, correlating high TAWSS to larger macrophage areas and necrotic core sizes. In addition, our study demonstrated new relations, correlating low OSI to larger macrophage areas, and a combination of low TAWSS and low OSI to larger cap thickness.

## Introduction

Atherosclerosis is a gradually progressing disease of the arteries characterized by vessel wall thickening due to the accumulation of lipids and inflammatory cells. This is referred to as plaque formation. Atherosclerosis is a multifactorial disease that can be aggravated not only by lifestyle factors, such as high caloric diet, physical inactivity, and smoking, but also by genetic factors. The initiation of plaque formation, however, has been strongly linked to a hemodynamic parameter: wall shear stress (WSS). WSS is the frictional force exerted by flowing blood on the vessel wall. Atherosclerotic plaques form at predetermined locations where WSS is low and/or oscillatory, which increases endothelial cell permeability and subsequent retention of lipoproteins (Lusis, 2000) and regulates pro-inflammatory signaling pathways in the endothelium, resulting in an increased influx of inflammatory cells (Gimbrone and García-Cardeña, 2013). The causal role of WSS in plaque initiation is evident (Kwak et al., 2014; Cunningham and Gotlieb, 2005); however, the influence of WSS on plaque progression is less clear. Initial plaque growth is generally accompanied by outward remodeling and preservation of low and/or oscillatory WSS levels. With disease progression, however, plaques will start to intrude the lumen, thereby affecting local hemodynamics. Upstream and at the throat of the plaque, WSS levels are high, while low WSS is found downstream (Slager et al., 2005). Based on their composition and the resulting risk of rupture, plaques are classified as stable or vulnerable. Vulnerable plaques are characterized by a thin cap, covering a large necrotic core (NC), and often present with high inflammatory activity and decreased smooth muscle cell content (Schaar et al., 2004). Intra-plaque hemorrhage (IPH) can also be present (Virmani et al., 2000). The morphology and composition of advanced plaques were shown to be inhomogenous, both in the axial and in the circumferential direction (Richardson et al., 1989; Dirksen et al., 1998; Burke et al., 1999; Wentzel et al., 2003; Cicha et al., 2011), which could indicate a relation between local hemodynamics and plaque composition. In addition, plaque rupture is most often encountered at the high WSS–exposed upstream site (de Weert et al., 2009). The interplay between WSS and plaque composition is a subject of debate: both low and high WSS have been linked to features of plaque vulnerability (Lovett and Rothwell, 2003; Cicha et al., 2011; Eshtehardi et al., 2012; Wentzel et al., 2012; Vergallo et al., 2014; Tuenter et al., 2016; Yamamoto et al., 2019). However, these studies differ in imaging modality used for assessing plaque composition, complicating one-to-one comparison of their results. In addition, studies of the carotid artery are often based on assumed WSS levels (Dirksen et al., 1998; Lovett and Rothwell, 2003; Fagerberg et al., 2010) or used animal models, which are not truly representative (Winkel et al., 2015; Daugherty et al., 2017). Since histology is the gold standard for the assessment of human plaque composition in high resolution, we previously developed a framework for accurate registration of MRI-derived WSS patterns to histological cross-sections (Moerman et al., 2019). In this study, we investigated the correlation between WSS and histologically identified components of plaque vulnerability in a dataset of advanced human carotid atherosclerotic plaques.

## Methods

### MR Imaging, Tissue Collection, and Histological Processing

MR imaging, tissue collection, tissue processing, and image registration procedures have been described in detail elsewhere (Moerman et al., 2019). In short, we imaged the carotid bifurcations of 11 patients scheduled for carotid endarterectomy (CEA) surgery in a 3.0-T MRI scanner (GE Healthcare, Milwaukee, United States). The MRI protocol consisted of a 3D black-blood fast spin-echo (3D-BB-FSE) sequence with variable flip angles (TR/TE: 1000/16 ms; FOV: 15 cm; slice thickness: 0.8 mm; matrix: 160 × 160; number of excitations 1; scan time: 190 s), which was optimized for visualizing lumen and outer wall geometry. CEA specimens were collected within 30 min after surgical resection, snap-frozen in liquid nitrogen, and stored at −80°C until *ex vivo* scans and histology were performed. Upon processing, CEA specimens were thawed, fixed in 4% formaldehyde, and immersed in PBS. *Ex vivo* MRI scans (T2w fast recovery FSE (frFSE); TR/TE: 2,500/66 ms; in-plane resolution: 0.1 × 0.1 mm; slice thickness: 0.5 mm; matrix: 256 × 256; scan time: ∼20 min; number of slices: 66) were performed using a 7.0-T MRI scanner (7.0T Discovery MR901, GE Healthcare, Milwaukee, United States).

CEA specimens were decalcified and cut into 1 mm thick consecutive axial cross-sections. “En face photos” were taken (IXUS 60, Canon, Tokyo, Japan) of the proximal side of each cross-section. The en face photos contained landmarks to facilitate registration of each en face photo to the photo of the adjacent cross-section, enabling reconstruction of a 3D stack of en face photos. After photographing, the 1-mm-thick axial cross-sections were embedded in paraffin. Each paraffin block was cut into consecutive 5 µm thick sections, which were processed for a series of histochemical staining procedures, that is, Miller’s elastic stain, hematoxylin–eosin, Martius scarlet blue, and Picrosirius red, as well as two immunohistological staining procedures for macrophages (CD68, Abcam, United Kingdom) and endothelial cells (CD31, Abcam, United Kingdom). For each tissue section, compositional characteristics of plaque vulnerability, that is, necrotic core (NC) and the IPH-associated protein fibrin, were delineated in a segmentation image (BioPix iQ3.2). This segmentation was based on the total set of histochemical stains. Based on the immunohistological staining for CD68, we made segmentation masks of the CD68-positive pixels by applying a color deconvolution filter in Fiji (Ruifrok and Johnston, 2001; Schindelin et al., 2012). Tissue areas containing artifacts and NC led to false-positive results and were removed from CD68-positive pixel selection.

### Computational Fluid Dynamics for Wall Shear Stress Calculation

Lumen contours of the stenosed bifurcations were delineated on the *in vivo* MRI scan using ITK-snap (Yushkevich et al., 2006) and exported as surface geometry VMTK (Antiga et al., 2008) was used for surface smoothing and centerline calculation. Clipping of inlet and outlets normal to the centerline was performed in ICEM (ANSYS ICEM 17.1, United States), and flow extensions were applied on the inlet and outlets using the VMTK. The length of the added flow extensions was five times the radius of the inlet or outlets. Subsequently, ICEM was used to generate a volume mesh containing on average 7 million elements. The volume mesh consisted of tetrahedral elements with five layers of prism elements at the wall. On the inlet of the common carotid artery (CCA), we applied a transient flow velocity profile using the theory of Womersley (Xu et al., 2018). This transient profile was based on the average flow waveform over one heart cycle and the heart rate reported by Lee et al. (2008). For each patient, the transient flow waveform was scaled to obtain an average WSS in the common carotid artery of 0.9 Pa (Lee et al., 2009) (Gnasso et al., 1997). As outlet boundary condition, the relative outflow to the internal carotid artery (ICA) and external carotid artery (ECA) was defined based on the stenosis degree (Groen et al., 2010). Blood density was set to 1060 kg/m^3^, and non-Newtonian fluid behavior was mimicked by the Carreau–Yasuda model using the parameters reported by Seo et al. (2005). The Navier–Stokes equations were solved numerically (ANSYS Fluent 17.1, United States) (convergence criteria: 1E-4 for continuity residual; 1E-5 for x-velocity, y-velocity, and z-velocity residuals; time step was 0.004 s) over 2 heart cycles. Time-dependent time-averaged wall shear stress (TAWSS) and oscillatory shear index (OSI) (Ku et al., 1985) were computed over the second heart cycle to account for initialization effects.

### Image Registration of MRI, CFD, and Histology

Image registration was performed according to the methods we described previously (Moerman et al., 2019). In short, *via* a series of rigid and nonrigid image registrations and transformations, the *in vivo* MRI lumen and its corresponding WSS map were transformed consecutively to the *ex vivo* and the en face image domain. Histology images were axially stacked and registered to the en face domain as well, resulting in co-registration of *in vivo* MRI-derived WSS and histology.

### Analysis and Exclusion Criteria

For a detailed description of data selection and analysis procedures, we refer to our previous publication (Moerman et al., 2019). In short, WSS patterns were axially averaged over −0.3 mm to +0.3 mm with respect to the axial location of the nearest histology section. Transversally, the WSS distribution over each lumen was discretized into eight radial bins (bin radius 45°). The points on the centerline of the transformed 3D WSS map were used as center points to create the radial bins. Plaque component measures were averaged in each radial bin. Per bin, we investigated the relation between local WSS and tissue composition of total intima depth. The dataset of 11 CEA samples yielded 183 axial cross-sections, of which 87 (48%) were included in the final analysis, yielding a total of 696 radial bins, of which 511 (73%) were included in the final analysis.

We excluded data, both axial cross-sections and radial bins, based on a set of criteria. Axial cross-sections were excluded if A) a part of the excised CEA lumen showed severe and nonuniform deformation with respect to the rest of the lumen, for example, large lumen collapse in the ICA compared to the CCA. The applied nonrigid lumen registration algorithms act on the global 3D geometry and are not able to correct for local severe tissue deformations; thus, accurate registration was impaired in such cross-sections. B) Lumen diameter on *in vivo* MRI was <3 pixels and/or showed a very large axial gradient. As discussed previously (Moerman et al., 2019), the applicability of our method should be carefully assessed in these cases since WSS calculations in highly stenotic areas are very sensitive to minor variations in lumen size, and a small mismatch in registration accuracy in regions with large axial gradients has a relatively large effect on the relation found between WSS and plaque composition. C) Low signal-to-noise ratio of the *in vivo* MRI images, which impaired reliable lumen segmentation. The radial bins were excluded based on the presence of 1) histological processing artifacts, 2) a mismatch in lumen registration between en face and histology images, or 3) a mismatch in lumen registration between *in vivo* MRI and en face images (Moerman et al., 2019). For the included radial bins, we quantified registration performance by calculating the average Hausdorff distance (HD) and dice similarity coefficients (DSCs) between en face and histology lumen segmentations and en face and *in vivo* MRI lumen segmentations (Moerman et al., 2019).

### Statistical Analysis

The range of absolute TAWSS and OSI values varied per artery. To define low, mid, and high WSS metric regions within each artery, patient-specific TAWSS and OSI tertiles were calculated. Statistical analysis was performed using a linear mixed effects model: WSS tertiles or OSI tertiles were set as a fixed factor and patient as a random factor. In addition, we tested the combination of WSS tertiles and OSI tertiles, including their interaction term, in one model to estimate the various parameters. Bonferroni correction was applied to adjust for multiple comparisons between WSS tertiles. The estimated means and standard error are reported. *P*-values < 0.05 were considered statistically significant.

## Results

### Registration Performance

The dataset of 11 CEA samples yielded 183 axial cross-sections, of which 87 (48%) were included in the final analysis. Ninety-six axial cross-sections were excluded; the majority of exclusions (59) were due to severe nonuniform deformation in the excised tissue or processing artifacts. Twenty axial cross-sections were excluded because the *in vivo* lumen was highly stenotic or had a large axial gradient, impairing accurate lumen segmentation for CFD simulations. Seventeen cross-sections were excluded because of bad signal-to-noise ratio on *in vivo* MRI. The 87 included axial cross-sections were divided into 8 radial bins, yielding a total of 696 radial bins, of which 511 (73%) were included in the final analysis. The majority of excluded radial bins (111) presented with histological artifacts. After the exclusion procedure, registration accuracy of the remaining dataset was quantified by calculating the dice similarity coefficient (DSC) and Hausdorff distance (HD) between the lumen segmentations of the en face and histology cross-sections and between the en face and the transformed *in vivo* cross-sections. For the histology-to-en face registration, we found a mean HD of 0.55 ± 0.07 (SEM) mm and a mean DSC of 0.85 ± 0.02 (SEM). The registration performance between *in vivo* MRI and en face was slightly better: the mean HD was 0.36 ± 0.06 (SEM) mm and the mean DSC was 0.91 ± 0.02 (SEM). These values were in the order of magnitude of previously reported similarity indices, describing registration of images of CEA specimens by multiple imaging modalities (Boekhoven et al., 2013).

### Wall Shear Stress Patterns in Advanced Carotid Atherosclerosis

**FIGURE 1:**
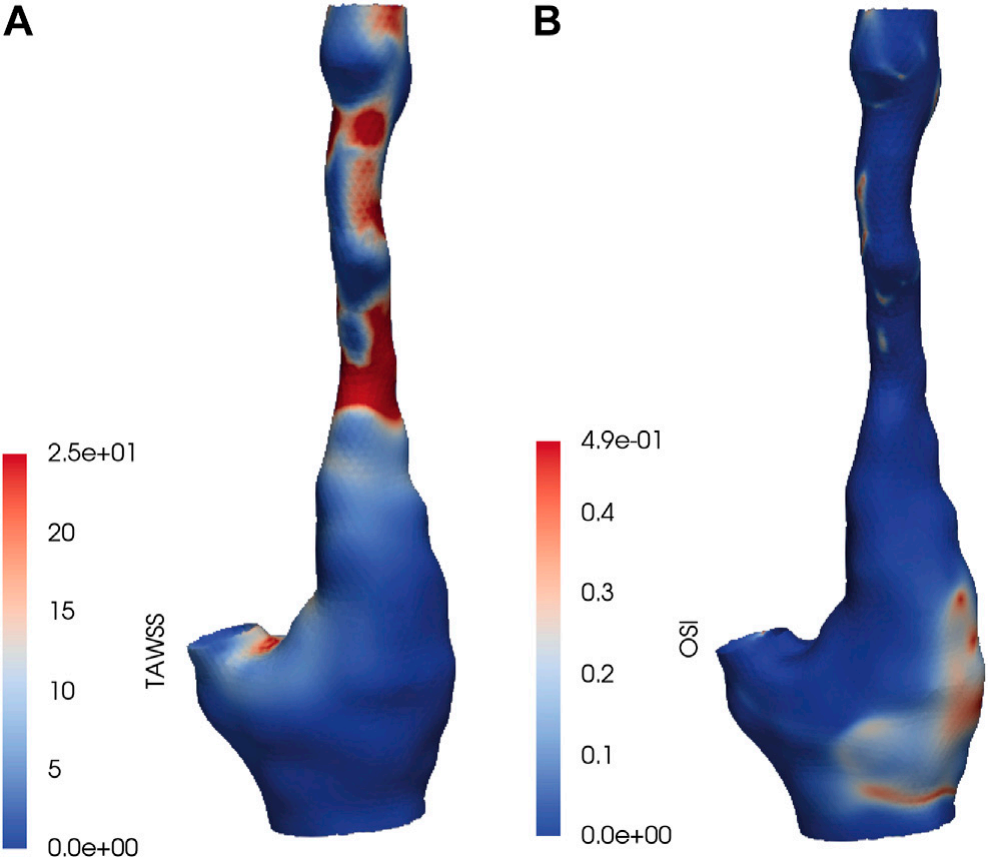
**(A)** Time-averaged wall shear stress (TAWSS) and **(B)** oscillatory shear index (OSI) values on a carotid bifurcation included in the dataset.

In Figure 1 the TAWSS and OSI patterns on a carotid bifurcation are shown. In all included carotid arteries, relatively high TAWSS was mainly located at the flow divider, at the ECA inlet, and upstream to and at locations of the maximally narrowed lumen. Low TAWSS was mainly found in the proximal CCA, at the lateral side of the carotid bulb, and at sites of relative lumen dilation. High OSI was generally found at the lateral side of the carotid bulb and downstream of stenoses, while low OSI was seen in relatively straight arterial segments, such as the proximal CCA, and at sites of lumen narrowing. The range of absolute TAWSS and OSI values varied per artery. After registration of the WSS maps to histology, WSS values were axially and radially averaged. In Table 1 the patient-specific tertile boundaries and the ranges of average TAWSS and OSI values, after registration and averaging per bin, are reported. In Table 2, the co-occurrences of TAWSS and OSI tertile values are reported. An inverse relation between TAWSS and OSI tertile values was most frequently encountered, but other combinations were also found.

**TABLE 1:** Patient-specific tertile boundaries and ranges of wall shear stress (WSS) metrics averaged on radial bins. TAWSS, time-averaged WSS; OSI, oscillatory shear index.

Patient	TAWSS tertile boundary low–mid [Pa]	TAWSS tertile boundary mid–high [Pa]	Range TAWSS—averaged over bins [Pa]	OSI tertile boundary low–mid [Pa]	OSI tertile boundary mid–high [Pa]	Range OSI—averaged over bins [Pa]
1	2.1	4.5	0.5–10.4	0.003	0.010	0–0.092
2	1.0	1.9	0.3–3.8	0.004	0.018	0–0.386
3	1.5	2.0	1.0–3.3	0.000	0.001	0–0.005
4	1.3	2.0	0.5–3.5	0.011	0.021	0–0.169
5	1.1	2.5	0.2–8.0	0.001	0.014	0–0.093
6	1.2	2.8	0.5–34.8	0.002	0.044	0–0.344
7	1.1	2.9	0.2–6.0	0.002	0.008	0–0.111
8	0.6	1.5	0.2–5.8	0.007	0.032	0–0.232
9	1.8	8.8	0.1–52.6	0.000	0.013	0–0.308
10	1.7	2.7	1.0–11.1	0.000	0.004	0–0.091
11	0.8	2.5	0.3–11.7	0.004	0.031	0–0.156

**TABLE 2:** Co-occurrence of TAWSS and OSI tertiles.

	Low OSI	Mid OSI	High OSI
Low TAWSS	11	38	120
Mid TAWSS	42	89	35
High TAWSS	116	39	14

### Histologically Determined Plaque Composition in Advanced Carotid Atherosclerosis

**FIGURE 2:**
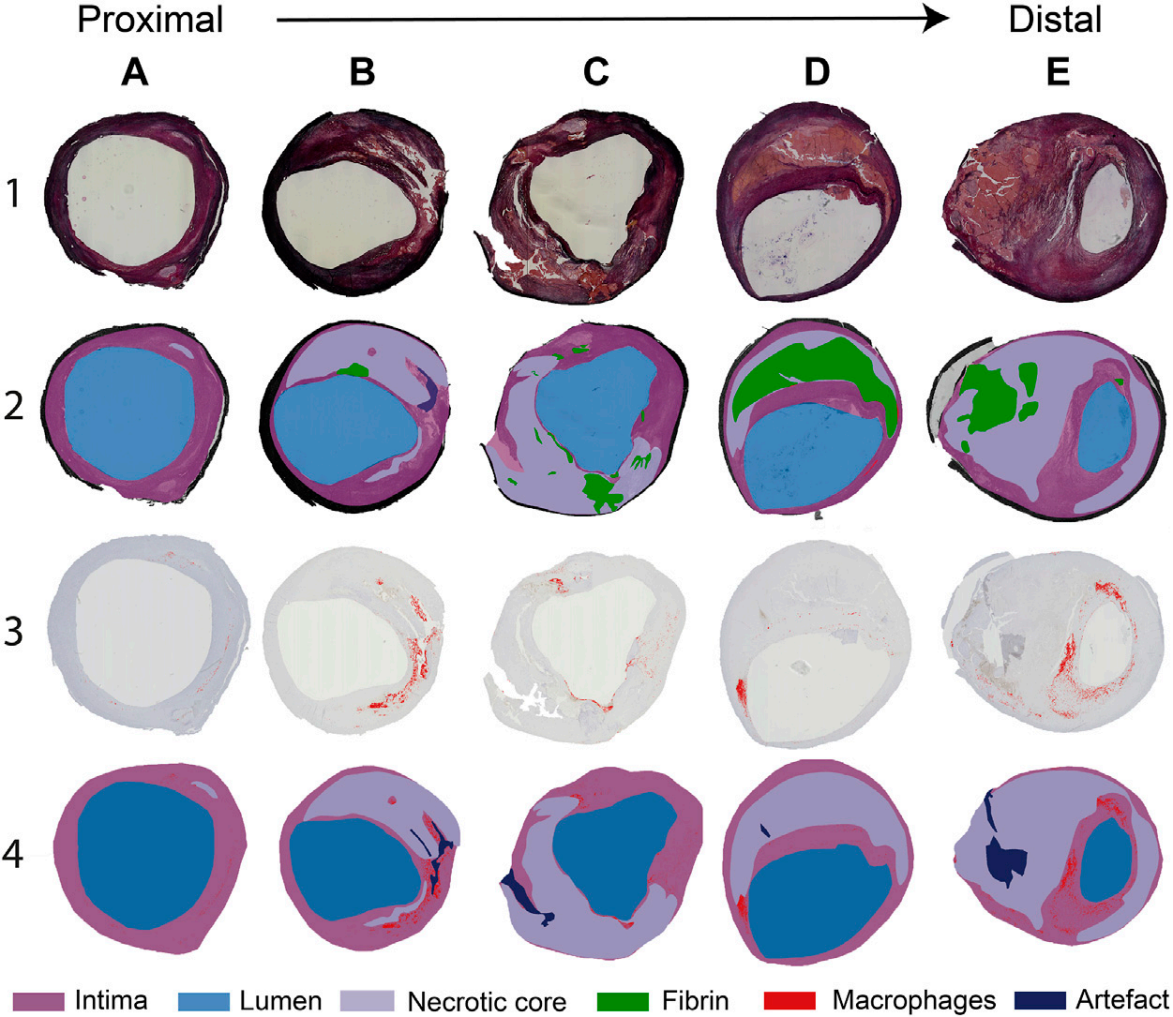
**(A–E)**. Selection of typical histology cross-sections of carotid plaques seen when moving from proximal to distal along the length of the carotid plaque. Cross-sections **(A–C)** are seen proximal to the flow divider and **(D,E)** distal to the flow divider. Row 1: Miller’s elastic stain. Row 2: segmentation of the necrotic core (NC), fibrin, and artifact, based on the combination of histochemical staining procedures performed. Row 3: CD68^+^ immunohistochemical stain. CD68-positive areas are marked in red. Row 4: segmentation of CD68^+^ stained slides, showing macrophage areas, NC, and artifacts.

In Figure 2, a selection of representative histological cross-sections is shown, along with the segmentation of lumen, fibrin, intima, NC, and macrophages. In general, histological cross-sections originating from the CCA showed a thickened intima with one or more NCs and small, elongated patches of macrophages. Both the NCs and the macrophages were generally located from the lumen up to a depth of half of the intima (Figure 2A). Relatively small fibrin areas could be observed as well. When moving from the proximal to distal region through the bulb, the plaque thickness varied in circumferential direction: plaque area and NC size were larger at the lateral side of the bifurcation (Figures 2B,C). Largest plaque area and eccentric plaque growth were observed in cross-sections harvested from the ICA (Figures 2D,E). Large NCs were present and showed large fibrin-positive areas. In these cross-sections, macrophages were generally distributed around the circumference of the lumen, were present in the cap, and were also seen deeper in the intima and at the edges of the NCs (Figure 2E). The amount of fibrous tissue was relatively low compared to proximal axial locations.

### Relation Between Wall Shear Stress Metrics and Plaque Composition

**FIGURE 3:**
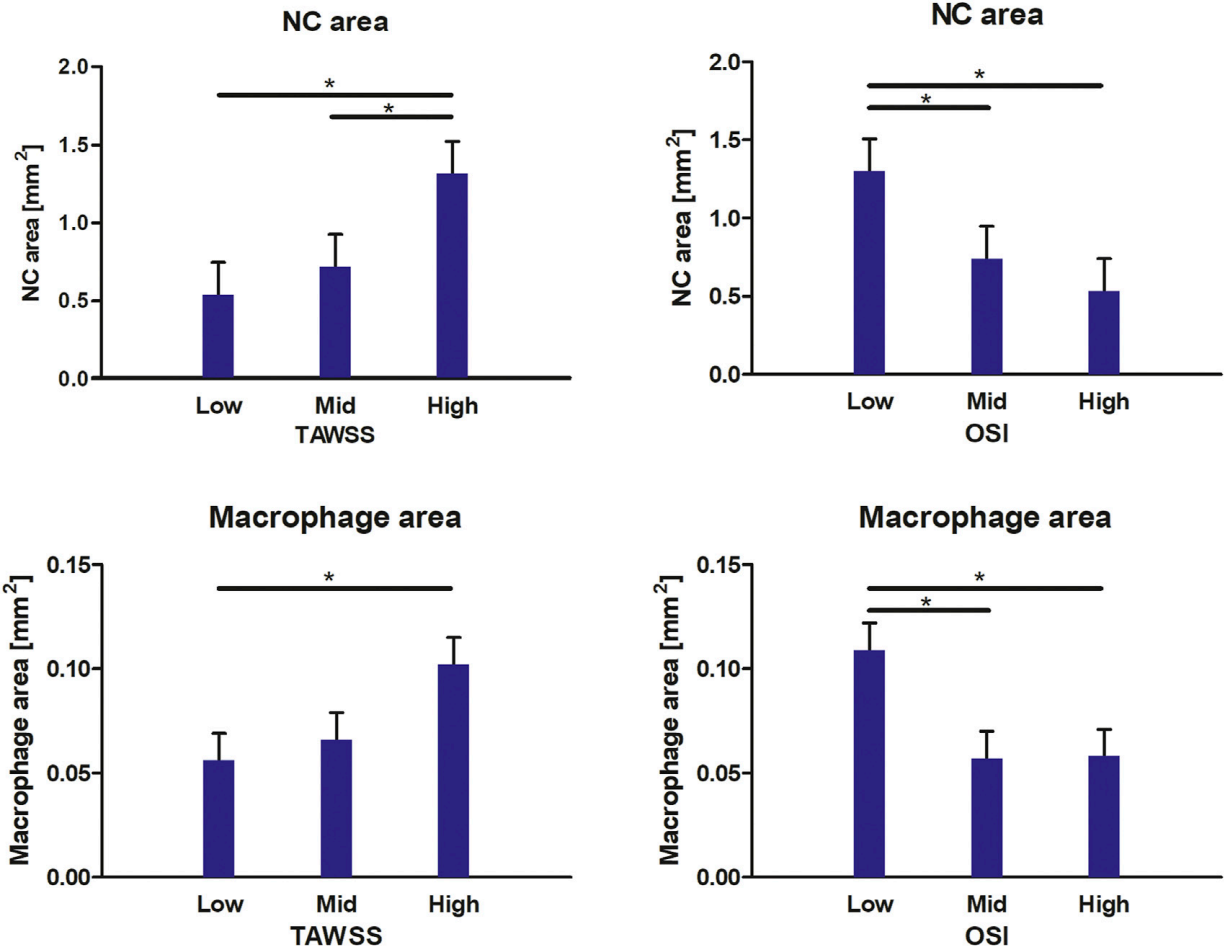
Relations between WSS metrics and plaque compositional characteristics. In high TAWSS-exposed regions and low OSI-exposed regions, a larger NC area and macrophage area are found. At low TAWSS and low OSI, a larger cap thickness is found. Data are presented as estimated mean +standard error. *p < 0.05.

For all included radial bins, we compared the average TAWSS and OSI levels, subdivided into patient-specific low, mid, and high tertiles, to the composition of the underlying plaque (NC area, fibrin area, macrophage area, and cap thickness). In Figure 3, the relations between WSS metrics and plaque composition are visualized. When considering the plaque composition of the total intimal area per radial bin, we found significantly larger NC areas in plaque regions exposed to high TAWSS, compared to low TAWSS (*p* = 0.000) and mid TAWSS (*p* = 0.004). In addition, we found significantly larger NC areas in plaque regions exposed to low OSI, compared to high OSI (*p* = 0.000) and mid OSI (*p* = 0.007). Regarding macrophage area, we found significantly larger macrophage areas in regions exposed to high TAWSS than in regions exposed to low TAWSS (*p* = 0.007). In addition, we found significantly larger macrophage areas in plaque regions exposed to low OSI, compared to high OSI (*p* = 0.002) and mid OSI (*p* = 0.002). When different combinations of TAWSS and OSI tertile values were analyzed, we found a significantly larger cap thickness in plaque regions exposed to both low TAWSS and low OSI, compared to low TAWSS and high OSI (*p* = 0.013). In the regions exposed to low OSI, a trend is observed for decreasing cap thickness with increasing TAWSS (low vs. mid TAWSS *p* = 0.076 and low vs. high TAWSS *p* = 0.060). No relations were found between the WSS metrics and fibrin area.

## Discussion

This was the first study that was able to make a direct comparison between local WSS metrics and histologically determined compositional characteristics of plaque vulnerability in a substantial number of human carotid plaques. The applied image registration pipeline allowed for a very accurate, localized assessment of this relation. We were able to take into account not just axial but also rotational matching of WSS and histology. Our cross-sectional analysis showed a relation between NC area and macrophage area with high TAWSS, as well as low OSI in advanced carotid atherosclerosis. Of these correlations, only a relation between presumed (because of the upstream location) high TAWSS and macrophage area was previously reported (Dirksen et al., 1998; Fagerberg et al., 2010). The relations between high TAWSS and cap thickness (Wentzel et al., 2013) in human coronaries and high TAWSS and IPH (Groen et al., 2007; Tuenter et al., 2016) in human carotids (Dirksen et al., 1998; Fagerberg et al., 2010), as previously reported, were not present in this dataset. When analyzing combinations of TAWSS and OSI tertile values, we found a significantly larger cap thickness in plaque regions exposed to both low TAWSS and low OSI, compared to low TAWSS and high OSI. The observed trend for decreasing cap thickness with increasing TAWSS in regions exposed to low OSI confirmed previous findings.^12,20^

### Wall Shear Stress Patterns in Advanced Carotid Atherosclerosis

The TAWSS patterns observed in this set of carotid arteries were in line with findings previously described in the literature (Ku et al., 1985; Kaazempur-Mofrad et al., 2004; Ladisa et al., 2010; Gallo et al., 2016; van Ooij et al., 2018; Vamsi Krishna et al., 2020). That is, low TAWSS was present at the CCA, at the lateral side of the carotid bulb, while high TAWSS was mainly seen at the upstream side of lumen stenoses. Generally, an inverse relation between TAWSS and OSI was found, as described before (Gallo et al., 2016). However, a subset of radial bins showed other relations between these two WSS metrics. As inflow boundary conditions, we did not use measured patient-specific flow profiles but applied estimated patient-specific flow profiles, scaled by inlet diameter, as we previously found that the absolute WSS levels in highly stenotic carotid arteries were affected mostly by the patient-specific geometry rather than the flow (Groen et al., 2010). Yet, large variations in WSS and OSI values were still encountered between patients, which stresses the large influence of geometrical factors on WSS patterns. Because of the large variation in WSS ranges between patients, it would be impossible to draw conclusions on the relation between WSS and plaque composition by using absolute WSS values. Therefore, we calculated patient-specific WSS tertiles. The use of relative instead of absolute WSS levels has the additional advantage of diminishing the sensitivity of the CFD results to the approximated boundary conditions that were applied.

### Histologically Determined Plaque Composition in Advanced Carotid Atherosclerosis

The histologically determined composition seen in this set of carotid plaques reflected histology results reported in the literature (Virmani et al., 2006b). That is, the greatest plaque cross-sectional area and smallest lumens were found in the ICA at cross-sections where the largest NCs were present, sometimes accompanied by fibrin-positivity, reflecting IPH. The majority of the excluded axial cross-sections and radial bins presented with processing artifacts, especially in the distal part of the plaque. The relatively “soft” necrotic and thrombotic tissue areas were more prone to deformation than tissue areas containing a higher amount of fibrous tissue. In addition, the tissue structure at areas that contained a high amount of calcium lost rigidity after decalcification, making these regions also susceptible to processing artifacts. The exclusion of these necrotic and calcified areas may have introduced a bias in our dataset toward moderately diseased tissue segments. The effect of this bias on the obtained results is unpredictable.

### Relation Between Wall Shear Stress Metrics and Plaque Composition

Our study was designed to assess the correlations between WSS metrics and compositional features of plaque vulnerability in carotid plaques. We found relations between TAWSS and OSI with NC area, macrophage area, and cap thickness. Regarding NC area, we found that NCs were significantly larger at locations exposed to high TAWSS. Besides the existence of a direct correlation between TAWSS and NC size, this finding might be explained by the fact that progression of atherosclerosis is accompanied by plaque growth and subsequent lumen intrusion. This local lumen narrowing will affect TAWSS patterns, inducing a local high TAWSS at the upstream side and at the throat of the stenosis. In addition, plaque area has been shown to be correlated to NC size (Wentzel et al., 2013; Ahmadi et al., 2015), a finding that links the co-existence of high TAWSS and NC size. A positive correlation between TAWSS and NC has been reported before in both carotid plaques (Yang et al., 2010) and in coronary plaques (Samady et al., 2011; Eshtehardi et al., 2012; Wentzel et al., 2013; Park et al., 2016). Similarly, we also found significantly larger NCs at regions exposed to low OSI. The OSI value describes how much the WSS vector deviates from its average direction. Thus, low OSI, indicating a relative constant WSS direction, is mainly expected at regions with high flow velocity and high TAWSS. Although a large NC size has been shown to be associated with rupture (Virmani et al., 2006a; Falk et al., 2013), based on this cross-sectional study, we cannot differentiate whether we are looking at a “true” relation between WSS-induced NC growth and vulnerability or simply at the effect of stenosis-induced altered hemodynamics. In addition to a larger NC area, we found significantly larger macrophage areas in plaque regions where OSI levels were low or TAWSS was high. Our findings confirmed the previously reported relation between presumed (because of the upstream location) high TAWSS and macrophage area (Dirksen et al., 1998; Fagerberg et al., 2010). To the best of our knowledge, we are the first to report a relation between low OSI and macrophage area. We found a new relation between cap thickness and WSS metrics when analyzing combinations of TAWSS and OSI tertile values. In plaque regions exposed to both low TAWSS and low OSI, cap thickness was significantly higher than in areas exposed to low TAWSS and high OSI. In addition, we observed a trend for decreasing cap thickness with increasing TAWSS in regions exposed to low OSI, confirming previous findings.^12,20^

### Implications of This Study

The relation between WSS and plaque vulnerability has been a topic of discussion for many years. Finding a direct association between WSS and risk of rupture might guide diagnostic approaches to identify the patient at risk or drive therapeutic development to stagnate disease progression. A combination of various study designs is necessary for finding evidence to reach these ultimate goals: both longitudinal studies, investigating the *in vivo* relation between WSS and plaque progression (Kumar et al., 2018; Stone et al., 2018); *in vitro* assays, investigating the pathways underlying the response of endothelial cells to WSS; and cross-sectional investigations, proving the existence and applicability of these relations.

The variability in results between previous studies and ours might be due to different reasons. First, differences in geometry, size, and/or origin of the vascular bed might influence the nature of the relation between plaque composition and a hemodynamic parameter, such as WSS. This, in combination with the generally small sample sizes in this kind of studies, increases the likelihood of varying outcomes. Second, the degree of disease progression is likely to influence findings in cross-sectional setups. As plaque histology could only be obtained from subjects who are eligible for an endarterectomy procedure, the study population involved elderly patients with advanced disease. As relations between WSS and plaque composition is dynamic and disease stage–dependent, these relations could be less obvious in our study population. Third, the study setup might be of influence. Only one previous study compared TAWSS and OSI to histologically determined vulnerability features in four CEA samples (Kaazempur-Mofrad et al., 2004) and found no correlations. In all other cross-sectional studies, plaque composition was determined by *in vivo* imaging, or relative WSS levels were assumed based on axial location instead of calculations. Finally, we studied relative differences in WSS parameters, not absolute levels, in a cross-sectional setup. With time, technological improvements regarding higher *in vivo* imaging resolutions might allow patient-specific flow data and high-resolution compositional information to be obtained. This in combination with a larger study number would be required to analyze whether WSS parameters can be used as a predictor of plaque composition and ultimately rupture risk.

In conclusion, our image registration pipeline can match histology to patient-derived WSS metrics and can be used to investigate the relation between these WSS metrics and features of plaque vulnerability. We found relations between TAWSS and NC area and between TAWSS and macrophage area, which were in agreement with the literature. To our knowledge, our study is the first to show significant relations between OSI and features of human plaque vulnerability. Unlike the general assumption, low OSI areas did not always coincide with high TAWSS. In fact, areas exposed to both low OSI and low TAWSS showed significantly thicker caps.

## Data Availability

The datasets presented in this article are not readily available because the data is generated in a consortium. Approval by the consortium is needed before the data can be shared. Requests to access the datasets should be directed to k.vanderheiden@erasmusmc.nl.
